# Modeling the temporal periodicity of growth increments based on harmonic functions

**DOI:** 10.1371/journal.pone.0196189

**Published:** 2018-04-25

**Authors:** José Angel Hidalgo-de-la-Toba, Enrique Morales-Bojórquez, Sergio Scarry González-Peláez, J. Jesús Bautista-Romero, Daniel Bernardo Lluch-Cota

**Affiliations:** 1 Centro de Investigaciones Biológicas del Noroeste (CIBNOR), La Paz, B.C.S., México; 2 Posgrado en Ciencias Marinas y Costeras, Departamento de Ciencias Marinas y Costeras, Universidad Autónoma de Baja California Sur, La Paz, BCS, México; University of Split, Faculty of science, CROATIA

## Abstract

Age estimation methods based on hard structures require a process of validation to confirm the periodical pattern of growth marks. Among such processes, one of the most used is the marginal increment ratio (MIR), which was stated to follow a sinusoidal cycle in a population. Despite its utility, in most cases, its implementation has lacked robust statistical analysis. Accordingly, we propose a modeling approach for the temporal periodicity of growth increments based on single and second order harmonic functions. For illustrative purposes, the MIR periodicities for two geoduck species (*Panopea generosa* and *Panopea globosa*) were modeled to identify the periodical pattern of growth increments in the shell. This model identified an annual periodicity for both species but described different temporal patterns. The proposed procedure can be broadly used to objectively define the timing of the peak, the degree of symmetry, and therefore, the synchrony of band deposition of different species on the basis of MIR data.

## Introduction

In marine fisheries resources (bivalve, fishes, cephalopods, etc.), the count of annual or daily growth increments in hard structures is used to estimate age and reconstruct growth rates [1–4]. This process necessitates age validation methods that confirm the increment periodicity of the analyzed structures (otoliths, shells, vertebrae, eye stalks, beaks, etc.). The marginal increment ratio (MIR) has been used for this purpose owing to its easy sampling requirements and low cost. According to Campana [5], the MIR is the proportion of the state of completion of the forming growth increment and the last completed growth increment, which ranges from 0%, when an increment is just beginning to form, to almost 100%, when a complete increment has formed (the proportion is less than 100%, because each new band tends to be slightly smaller than the previous fully formed one). During the band formation process, after a growth increment of a single individual has been completed, the MIR will immediately plunge to near 0% as a new growth increment begins to form; a graph of MIR data thus follows a sawtooth shape. In a population, this shape changes based on the degree of asynchrony of the growth increment formation between the individuals of the sample analyzed to a sinusoidal cycle, as periodic accretion of calcium carbonate is controlled by biological clocks, and growth rates are sensitive to environmental conditions (e.g., temperature, food, salinity, and pollution) [6] and individual ontogeny [7].

Despite the usefulness of the MIR as a validation tool, its use has frequently been limited to the graphical representation of observed trends, and thus, it has been lacking a precise measurement of the periodicity of the growth increments [8,9]. Furthermore, statistical analysis of the MIR would allow additional analysis of the data, like comparisons between the timing of the growth increments of different species/stocks or with environmental data that might be driving the progression of the growth increments. In this regard, several efforts have focused on improving the analysis and interpretation of monthly MIR values, which include the use of parametric and non-parametric ANOVA [10,11]. Although both these statistical procedures facilitate the identification of significant monthly or seasonal differences throughout the year, they are unable to estimate either a cyclical pattern in the data or its duration. In the first attempt at such modeling, Okamura et al. [12] reported the identification of cyclical patterns by modeling MIR data in age validation studies. These authors used MIR data of the vertebrae of the Alaska skate (*Bathyraja parmifera*), the measurements of which were re-scaled to radians and analyzed in a circular–linear regression with random effects using a truncated wrapped Cauchy distribution as an objective function. More recently, the MIR data of geoduck clams (*Panopea generosa* and *Panopea globosa*) were modeled using a sinusoidal function parameterized through a maximum likelihood estimator, with confidence intervals being estimated from Monte Carlo simulations [8].

These statistical procedures assumed that the data followed a cyclical pattern; however, the MIR is based on the proportions of growth increments that are irregular throughout the year, as a consequence of different environmental and biological factors, e.g., water temperature, food availability, and spawning [5,13–15], and thus, MIR progression would be irregular throughout the year on a monthly basis. Under these conditions, the inclusion of harmonic functions within the set of candidate models can allow the analysis of the total variation of the observed data, providing an additional hypothesis to the models previously proposed.

In this study, we propose a method of analysis that can be used to validate and estimate growth band deposition, using *P*. *generosa* and *P*. *globosa* MIR data for illustrative purposes. Our methodology incorporates the following improvements to previous contributions: (i) implementation of a multi-model analysis comparing single and second order harmonic functions, and (ii) parameter estimation through an improved normal distribution-based likelihood function; it represents a more simplistic mathematical expression, increasing its performance. This objective function is the best maximum likelihood function used for parameter estimation [16,17].

## Materials and methods

### Data origin

In this study, we used MIR data published by Bautista-Romero et al. [8]. The samples were collected from monthly commercial captures of two geoduck species on the west coast of the Baja California Peninsula. *P*. *generosa* was collected at Punta Canoas (29°25′N, 115°12′W) and *P*. *globosa* at Bahía Magdalena (24°35′N, 112°00′W). Permission to use these commercial fisheries data was granted by the Mexican federal government (Comisión Nacional de Acuacultura y Pesca) through a scientific collecting permit number DGOPA-10885-091006-4963 with fishermen of the Sociedad Cooperativa Pescadores de Altamar y Acuícola Bahía Santa Maria SCRL in Bahía Magdalena, as well as through biological samples provided by Dr. Alma Rosa García Juárez of the Centro Regional de Investigación Pesquera in Ensenada with the logistic support of fishermen of the Sociedad de Producción Rural Punta Canoas SRLCV in Punta Canoas. For details on the capture method and sample processing, see Hidalgo-de-la-Toba et al. [18] and González-Peláez et al. [10]. Growth increments in the shell of *Panopea* species are delimited by distinctive growth marks that are deposited periodically. The MIR was measured as the percentage between the last growth increment (MI; the distance between the last growth mark and the edge of the hinge plate section) and the penultimate growth increment in the shell (PI; the distance between the last and penultimate growth marks):

MIR=MI/PI×100% (1)

### Variability in growth increments

To establish if the growth increments among different ages of geoducks were similar among them, an analysis comparing the tendencies in the growth increment ratio for each age class (*IR_i_*) was done according to the following procedure: (a) for each species, a random subsample of 30 individuals was selected and a digital image taken from a cross-section of the whole hinge plate region. The growth increments of each cross-section were measured using the software SigmaScan Pro (version 5.0.0) following the direction of growth. Thus, *IR_i_* = *GI_i_*/*GI*_*i*−1_ × 100%, where *GI_i_* is the relative size of the growth increment at age *i*, and *GI*_*i*−1_ is the relative size of the previous growth increment with respect to age *i*; (b) a linear regression (α = 0.05) was fitted to test whether the *IR_i_* (dependent variable) increased or diminished with age (independent variable). This was done starting at the age of the youngest individual used in the MIR modeling (5 years old for *P*. *generosa* and 7 years old for *P*. *globosa*).

### MIR periodicity modeling

In this study, MIR periodicity was modeled using harmonic functions. These functions have been applied in fisheries studies assuming symmetrical curves, e.g., individual growth models incorporating a seasonal oscillation into the von Bertalanffy growth function [19–21], reproductive processes such as gonadosomatic condition [22–24], and egg production associated with seasonal trends [25]. These functions have also been applied to study the community dynamics of small fishes in freshwater wetlands [26], time-series of bottom trawl fisheries for analyzing the optimal harvest [27], and population dynamics based on an age-structured deterministic model [28]. Accordingly, we used two harmonic functions that are commonly employed and referred to in the literature: single order (H1, Eq 2) and second order (H2, Eq 3) [25], [29]:

$$\psi_{e,i}=\beta_{0}+\beta_{1}sin\;\left(2\pi \frac{t_{i}}{\gamma }\right)+\beta_{2}cos\;\left(2\pi \frac{t_{i}}{\gamma }\right);$$ (2)

$$\psi_{e,i}=\beta_{0}+\beta_{1}\sin\;\left(2\pi \frac{t_{i}}{\gamma }\right)+\beta_{2}\cos\;\left(2\pi \frac{t_{i}}{\gamma }\right)+\beta_{3}sin\;2\;\left(2\pi \frac{t_{i}}{\gamma }\right)+\beta_{4}cos\;2\;(2\pi \frac{t_{i}}{\gamma });$$ (3)

where ψ_*e*,*i*_ represents the MIR estimated by the harmonic model, *γ* represents the time unit in which events were measured (a value of 12 corresponds to a monthly scale in an annual cycle); *t*_*i*_ is the month number when the samples were collected; and *β*_n_ are constants that express the amplitude in the model.

The H1 is considered symmetrical because the time for the minimum ψ_*e*,*i*_ is one-half cycle before or after the time of the maximum ψ_*e*,*i*_. Meanwhile, the H2 proposed here adds higher harmonics through Fourier analysis, which reduces the variance of the data by fitting an irregular curve. In this way, MIR data that lack a symmetrical relation could be explained [29].

### Parameter estimation

Following the recommendations of Campana [5] and Okamura et al. [12], the observed MIR data(ψ_*o*_) were analyzed over two repeated periods of annual variation. Model parameters were estimated by maximum likelihood, which consists of maximizing expression [17]:

$$-2\;\ln\left(L\right)=n\;\left[ln\;(2\pi \frac{\sum_{i=1}^{n}{\left({ln\psi }_{o}-{ln\psi }_{e}\right)}^{2}}{n})+1\right]$$ (4)

where *n* is the number of observations. A multiplicative error was used to stabilize the variance [(*ln* ψ_*o*_−*ln* ψ_*e*_)^2^]. The search for the *θ*_*i*_ parameters was realized by maximizing the function -2*ln*(L) using the Newton algorithm [30].

### Likelihood intervals

According to Pawitan [31], in some statistical problems, it can be difficult to interpret the observed likelihood function directly to communicate the uncertainty regarding *θ* parameters. However, the likelihood intervals are a useful supplement to the maximum likelihood estimator, acknowledging the uncertainty in the *β*_n_ parameters, defined in this procedure as *θ* parameters; hence, it is simpler to communicate the uncertainty than to determine only the final value of the objective function (4). Hence, the uncertainty for the *θ* parameters in both harmonic functions was estimated as follows:

$$\left\{\theta ,-2ln\frac{L(\hat{\theta })}{L(\theta )}\leq 3.84\right\}$$ (5)

where $L(\hat{\theta })$ is the maximum likelihood estimator; L(θ) represents the hypothetical values of the *θ* parameters assumed through the likelihood profile; and 3.84 is the critical value for a *χ*^2^ distribution with *df* = 1, and 100 × (1 − *α*)% confidence region for each *θ* parameter [32,33]. Consequently, the likelihood intervals were used to estimate the uncertainty in the *θ* parameters. This procedure was implemented in Visual Basic Application ver. 6.0™.

### Model selection

Since H1 is a model nested in H2, the model selection was based on a likelihood ratio test (LRT), expressed as follows:

$$LRT=2[L(\psi_{o}|H1)-L(\psi_{o}|H2)].$$ (6)

LRT calculates how many times more likely the data are under the H1 model versus the H2 model, with the null hypothesis H_o_: $L(\psi_{o}|H1)=L(\psi_{o}|H2)$. Under the null hypothesis, the test statistic converges asymptotically to the *χ*^2^ distribution with *df* = 1, *α* = 0.05.

## Results

**Fig 1:**
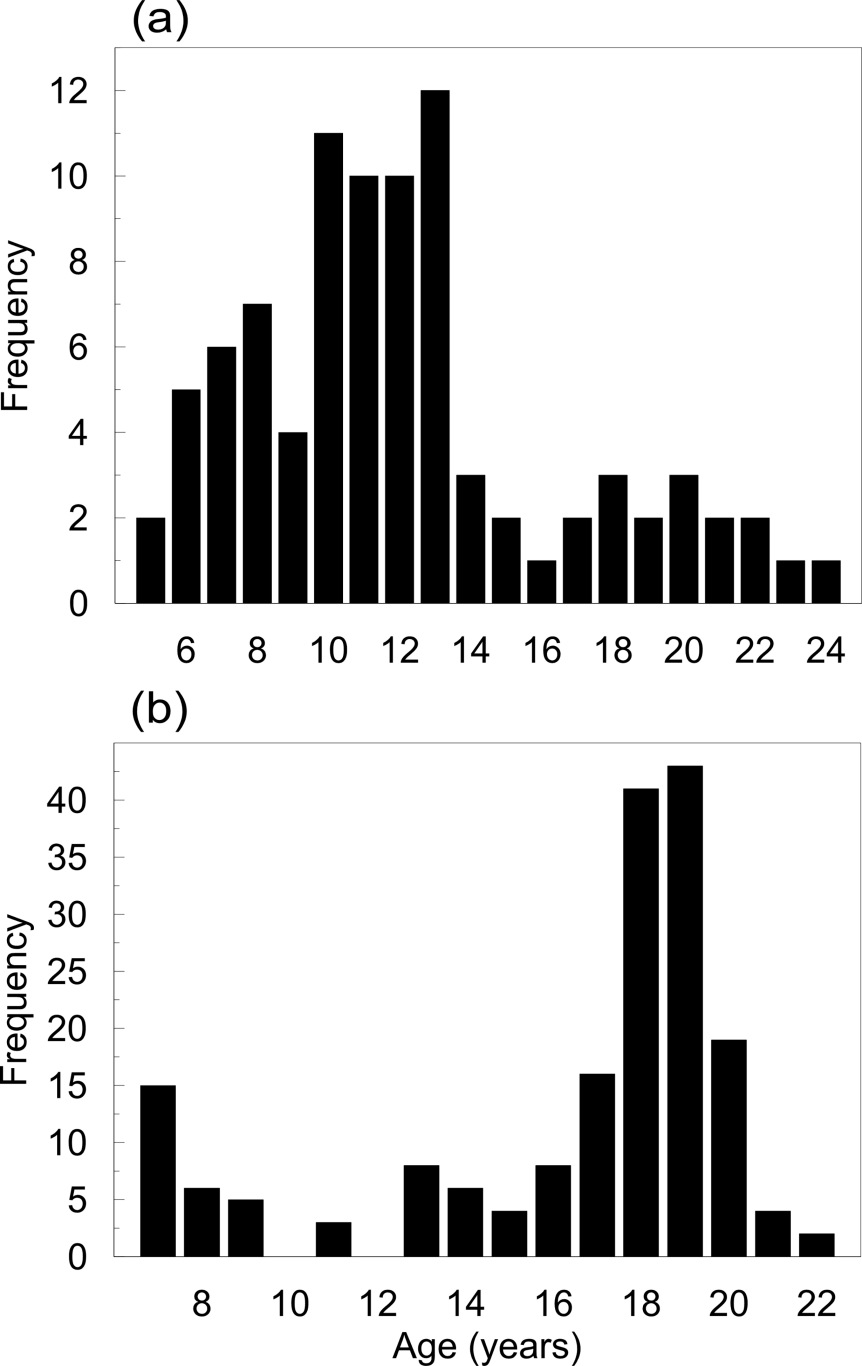
Age frequency distributions. (a) Age frequency for *Panopea generosa* from Punta Canoas. (b) Age frequency for *Panopea globosa* from Bahía Magdalena.

**Fig 2:**
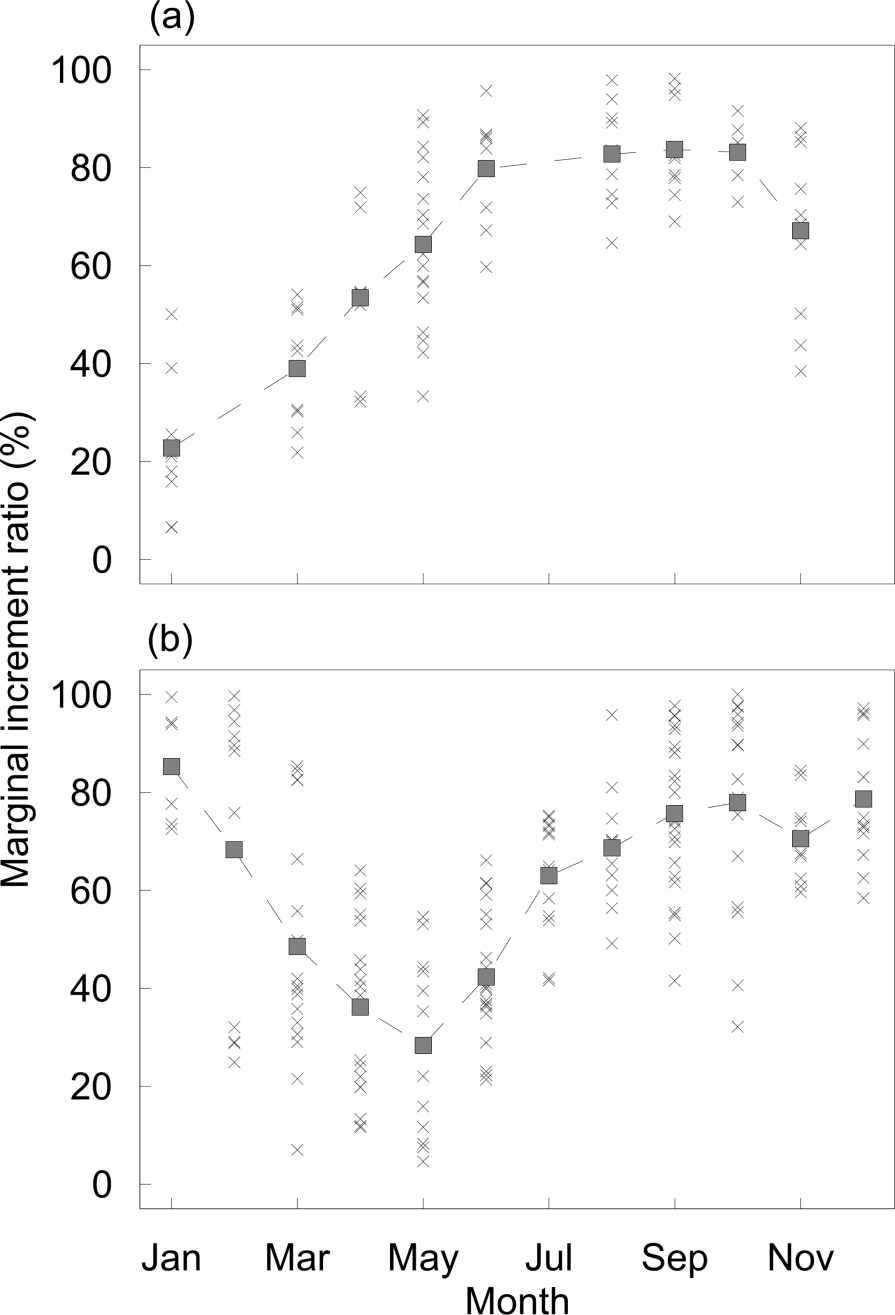
Marginal increment ratio data. (a) Marginal increment ratio (MIR) data for *P*. *generosa* from Punta Canoas. (b) MIR data for *P*. *globosa* from Bahía Magdalena. The square (■) indicates the monthly mean.

MIR data from 89 individuals of *P*. *generosa* (Punta Canoas) from 5 to 24 years old (Fig 1A) and 183 individuals of *P*. *globosa* (Bahía Magdalena) from 7 to 22 years old (Fig 1B) were analyzed. The monthly average and general trend of the data are shown in Fig 2. For both species, a sinusoidal pattern in the time-series was observed. These patterns showed that for *P*. *generosa*, the average MIR values for January were lower than the values estimated from June to October. Conversely, in January, *P*. *globosa* showed its highest MIR average, which decreased to a minimum value in May.

**Fig 3:**
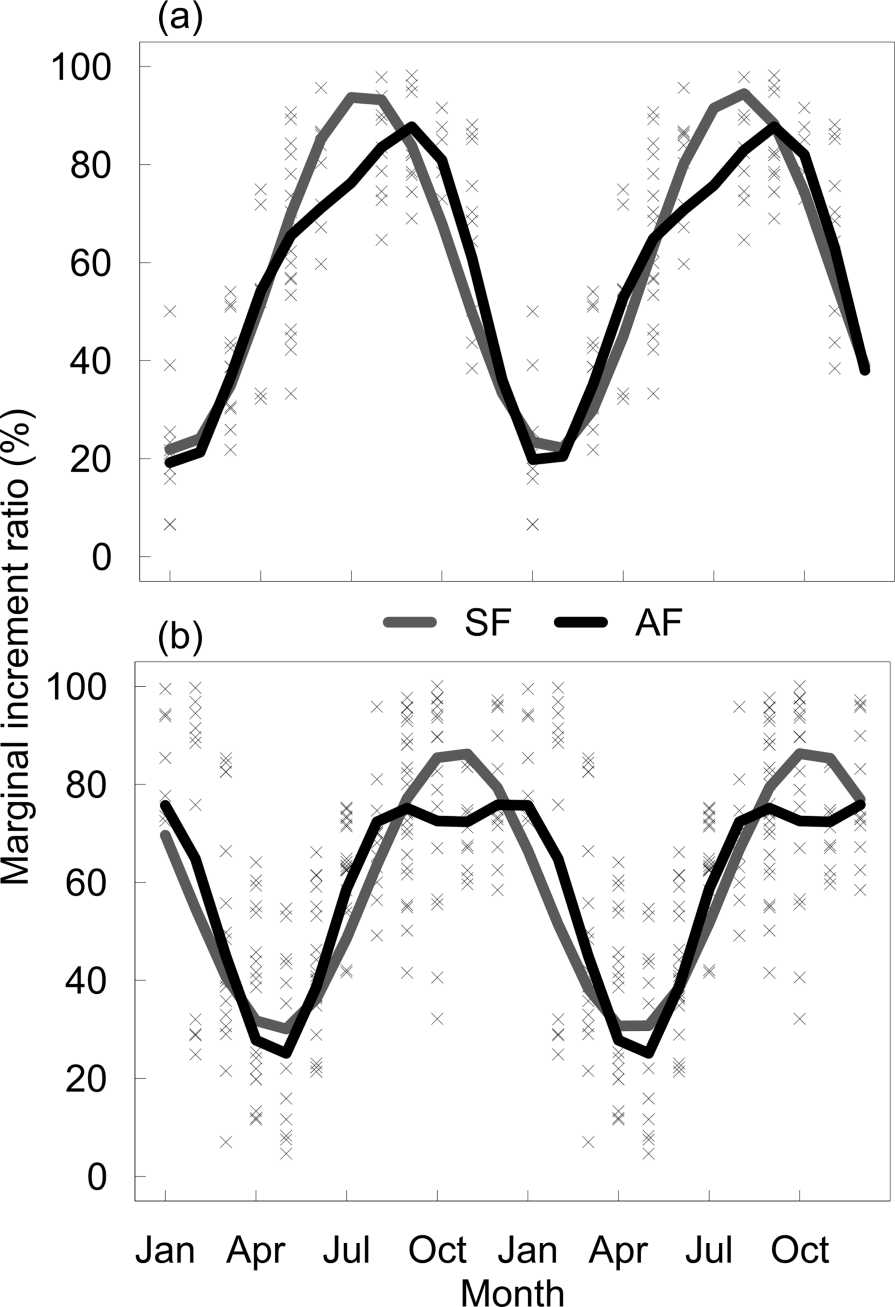
Harmonic models fitted to the MIR data. (a) Harmonic models fitted to the MIR data of *P*. *generosa* from Punta Canoas. (b) Harmonic models fitted to the MIR data of *P*. *globosa* from Bahía Magdalena. H1 = single order harmonic function; H2 = second order harmonic function.

The two harmonic functions were fitted to the ψ_o_ of *P*. *generosa* and *P*. *globosa* (Fig 3). The single order model described a sine curve for *P*. *generosa*, in which band formation began in January and reached a maximum in August. In the case of *P*. *globosa*, band formation was estimated to begin in May and reach a maximum in November. In contrast, the second order model showed that the band formation in *P*. *generosa* began in January and exhibited an inflection in its slope during May, indicating that band formation is slower at this time. Accordingly, the maximum ψ_e_ is reached during September, a month later than the maximum time estimated using the H1 (Fig 3A). In the case of *P*. *globosa*, H2 also identified May as the beginning of growth band formation, where the maximum ψ_e_ is reached during August, although similar values were estimated until January. Accordingly, a flattened dome pattern was observed, with a high ψ_e_ for 6 months (Fig 3B), suggesting a broad period of band formation. The parameters and CI for the models fitted to the ψ_o_ of *P*. *generosa* and *P*. *globosa* are shown in Table 1. The parameter *γ* for H1 has values close to 12 months (12.16 for *P*. *generosa* and 11.82 for *P*. *globosa*), suggesting an annual periodicity. The *γ* parameter for H2 also showed a similar periodicity (12.08 for *P*. *generosa* and 12.00 for *P*. *globosa*).

**Table 1:** Parameters of the harmonic models fitted to the marginal increment ratio (MIR) data of *Panopea generosa* from Punta Canoas and *Panopea globosa* from Bahía Magdalena.

Species	Model	Parameter	Value	Lower LI^§^	Upper LI^§^
*P*. *generosa*	H1*	*β*_0_	58.10	56.62	59.66
		*β*_1_	-21.83	-23.86	-19.74
		*β*_2_	-29.30	-31.48	-26.98
		*γ*	12.37	12.26	12.48
	H2**	*β*_0_	57.55	56.24	58.93
		*β*_1_	-25.36	-27.35	-23.29
		*β*_2_	-18.54	-20.28	-16.69
		*β*_3_	-8.40	-10.16	-6.54
		*β*_4_	-4.80	-6.82	-2.79
		*γ*	12.08	11.99	12.18
*P*. *globosa*	H1*	*β*_0_	58.31	56.85	59.82
		*β*_1_	-16.85	-18.93	-14.71
		*β*_2_	23.05	20.91	25.11
		*γ*	11.78	9.87	13.70
	H2**	*β*_0_	58.66	57.32	60.07
		*β*_1_	-15.07	-16.98	-13.09
		*β*_2_	18.59	16.60	20.50
		*β*_3_	10.63	8.65	12.54
		*β*_4_	-1.45	-3.38	0.51
		*γ*	12.00	11.90	12.09

*H1 = single order harmonic function

**H2 = second order harmonic function

^§^LI = likelihood interval

For *P*. *generosa*, twice the difference in the negative log-likelihood between the H1 and the H2 was 53.13. For *P*. *generosa*, this difference was 64.73. In both cases, the chi-square probability of change with two degrees of freedom had *P* < 0.05; the two sinusoidal functions were significantly different based on the likelihood ratio test (Table 2).

**Table 2:** Candidate models showing the number of parameters (*θ_i_*), likelihood value (−2ln(L)), number of parameters (*θ_i_*), LRT results, and *χ^2^* value.

Species	Model	*θ*_*i*_	−2ln(L)	LRT	*χ*^2^
*Panopea generosa*	H2**	6	82.53	53.13	3.84
	H1*	4	109.09		
*Panopea globosa*	H2**	6	388.68	64.73	3.84
	H1*	4	421.04		

*H1 = single order harmonic function

**H2 = second order harmonic function

**Fig 4:**
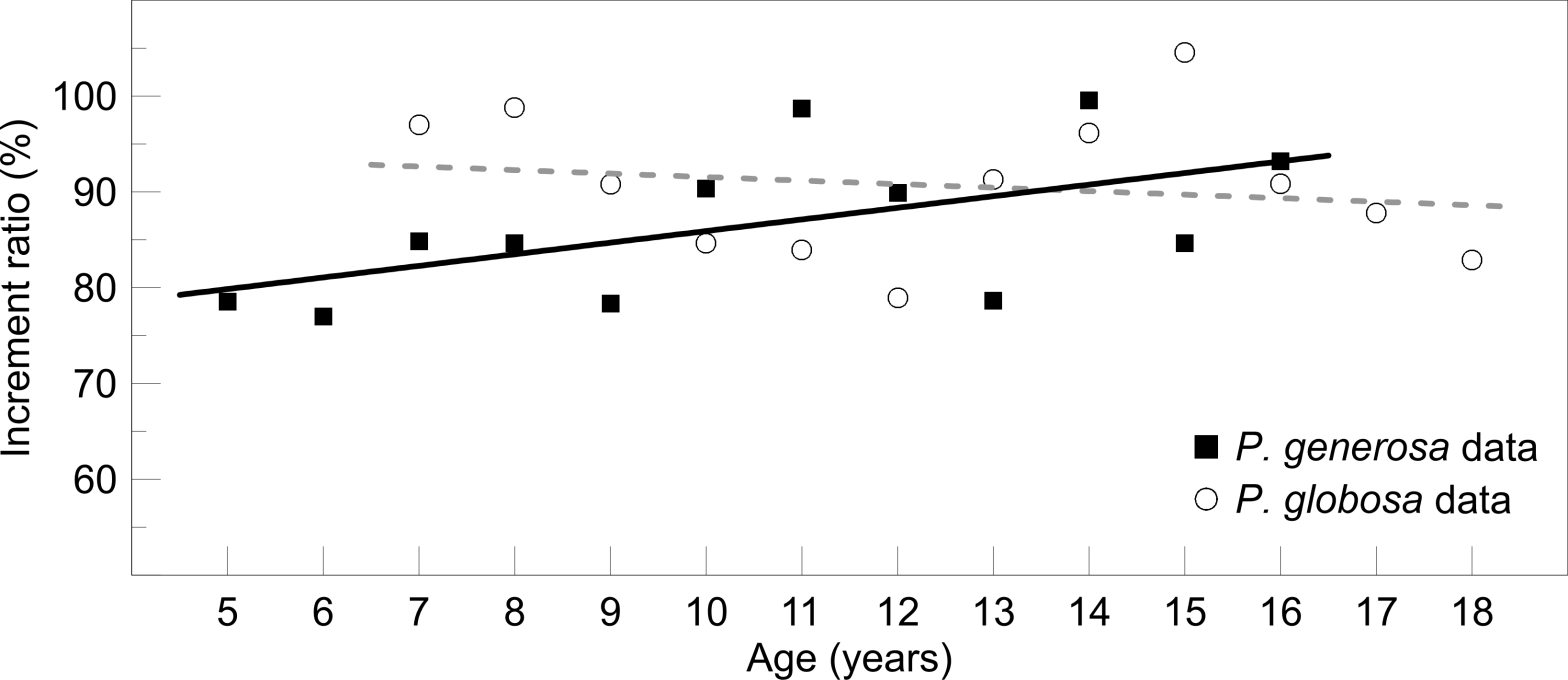
Tendency of the growth increment index data. Linear regression applied to the average growth increment ratio grouped by age. The black line is for *P*. *generosa* from Punta Canoas, and the grey dashed line is for *P*. *globosa* from Bahía Magdalena.

The results of the variability in growth increments explained that there was no effect of age on the IR_*i*_, implying that the marginal increments were similar through the age groups analyzed for each species. When the linear models were fitted to the data, they did not show interdependence between IR_*i*_ and age groups, where the correlation coefficients for *P*. *generosa* and *P*. *globosa* were r^2^ = 0.24 (*F*_*1*,*10*_ = 4.44; *P =* 0.06) and r^2^ = -0.07 (*F*_*1*,*10*_ = 0.32; *P* = 0.58), respectively (Fig 4).

## Discussion

### MIR data modeling as an age validation method

Considering that the natural pattern of MIR progression is a sinusoidal cycle [5], we here proposed the modeling of the temporal periodicity of growth increments that contrasted single and second order harmonic functions based on information theory, which is a strong statistical approach for model selection in ecology and fisheries analyses [34,35]. Specifically, in this study, model selection was carried out by employing the likelihood ratio test, which is widely used to compare the performance of nested models [36,37].

In recent years, the modeling of MIR data has been undertaken to improve its utility as an age validation method. Okamura and Semba [38] proposed a binomial model of marginal increments based on the presence/absence of opaque bands in the vertebrae of the shortfin mako shark (*Isurus oxyrinchus*), which was linked with a von Mises distribution for circular data and involved a transformation to radians showing symmetrical cycles that expressed annual periodicity. This method cannot be directly applied to MIR data, because binary data are required for its conception. However, MIR data can be used as auxiliary information for defining the presence of the opaque band. Wakefield et al. [39] used this approach to validate the annual periodicity in sagittal otoliths of a snapper (*Chrysophrys auratus*). Thereafter, the first approach that directly modeled MIR data was proposed by Okamura et al. [12], in which the time (months) transformed to radians was used to estimate the annual periodicity of growth bands in the vertebrae of the Alaska skate (*B*. *parmifera*). Modeling of the data was performed through a circular–linear regression with random effects using a truncated wrapped Cauchy distribution as an objective function. To compare annual cycles, three hypotheses were assumed: (i) a no-cycle model, (ii) a one-cycle model, and (iii) a two-cycle model. Independently of the hypotheses, in its conception, these were modeled through a symmetrical cycle, and the periodicity was forced to exactly 12 (for the one-cycle model) or 6 months (for the two-cycle model). Recently, Bautista-Romero et al. [8] used a normal distribution-based likelihood function, fitting a symmetrical sinusoidal model to the MIR data of two species of geoduck clams and assuming additive residual errors. In this model, the time was not transformed, and the periodicity was not initially fixed because it was calculated from the estimated parameters. The model indicated that both species had an annual cycle of growth band formation.

In the present study, the parameters were estimated according to an improved normal distribution-based likelihood function (−2ln(L)). By increasing the fit of the second order harmonic model to data that contained irregularities, the band growth formation may have an extended duration, or it may follow a slow and continuous increment. Given the dispersion commonly observed in MIR data, a multiplicative error was used. In contrast to additive errors, multiplicative errors allow the stabilization of variance [40]. The periodicity (γ) in the candidate models was not fixed, being estimated by the fitting procedure. By ensuring that the cycle is freely estimated by the model, we avoid the subjectivity of fixing the periodicity of the data. Comparatively, in previous studies, the temporality that defines the beginning of the symmetrical cycle depends on the intersection with the abscissa estimated in the circular–linear model. Given that the abscissa is pre-defined and expressed in radians, it represents a fixed value, and consequently, only the slope is calculated by the circular–linear model [12,38]. This indicates that, given that this is a straight line with a constant trajectory, it does not permit biological interpretation, thereby limiting the possibility of explaining changes in the pattern of the growth increments as a consequence of environmental and biological effects.

Considering that MIR data are highly variable, the presence of outliers has implications for the performance of an objective function and may result in biases in parameter estimation [41,42]. This could consequently affect the results of the selection criteria. Okamura and Semba [38] and Wakefield et al. [39] used only the Akaike information criterion (AIC) to determine the periodicity of growth bands. This criterion appears to be statistically insufficient because the AIC has poor performance when there is an over-dispersion in the data, which is a feature of binary data. Under these conditions, it is preferable to use the AIC for over-dispersed count data (QAIC). This criterion makes a quasi-likelihood correction when data are over-dispersed by including the term $\hat{c}$. If $\hat{c}$ = 1, then no over-dispersion exists; otherwise, if $\hat{c}$ is > 4, then it is assumed that a structural lack of fit is probably entering the estimate of over-dispersion [36]. Okamura et al. [12] used the AIC to identify both the month during which the cycle begins and its periodicity. The implementation of the AIC was unnecessary in the first case, because the model formulation was the same, and only one parameter needed to be estimated. On the basis of fit and parsimony, in situations where the candidate models share the same number of parameters, the goodness of fit is sufficient to determine the best hypothesis [36].

In summary, our proposed methodology has the following attributes compared with previous modeling of MIR data: (i) it is computationally more efficient because MIR data are not transformed or re-scaled, and the objective function is based on a normal probability density function; (ii) it has versatility for identifying regular and irregular patterns in the MIR data; and (iii) it enables the estimation of the duration of a cycle without depending on previously defined periodicities.

### Age validation in *Panopea* spp

Growth increment patterns in hard structures such as seashells and otoliths have been used in both the reconstruction of environmental and ontogenetic events and in the estimation of age data [13], where water temperature, food availability, and spawning events have been suggested as possible factors influencing growth band formation [5,43]. Specifically, for *Panopea* spp., the growth increments are considered integrated measures of metabolic activity over time and are influenced by food availability, temperature, and disturbances associated with anaerobic metabolism caused by deep-burrowing, the temporality of which indicates the presence of climate signals [14,15,44,45].

Several methods of validation of the annual deposition of growth lines have been used for different species of geoduck clam: (a) known age: a single cohort of *Panopea zelandica* was followed during a predefined number of years by counting the growth lines in shells of known age [46]; (b) mark-recapture: for *P*. *generosa*, the annual deposition of internal growth lines was experimentally confirmed through mark-recapture, as well as by the comparison of the age structures of individuals from impacted and pristine areas [44,47]; (c) cross-dating: a dendrochronological approach was used for *P*. *generosa* [15,48] and *P*. *globosa* [49]; (d) marginal increment analysis (MIA): a qualitative method based on the location of the growing edge in relation to the last completed growth line in *P*. *generosa* [44] or an alternative method based on the degree of transparency of the border ring of *Panopea abbreviata* [50], and a quantitative method (MIR) that compares the distance between the hinge plate margin and the last growth line versus the penultimate increment measured as the thickness of the last complete band [10]; and (e) radiochemical dating: annual growth line deposition has been validated directly via isotopic carbon signatures of ^14^C:^13^C ratios incorporated into the valves of *P*. *generosa* after 1957 [51,52].

In the present study, we found that for both the examined species, the second order harmonic model was the best candidate model for confirming annual periodicity. Nonetheless, the trajectories of the models fitted to the data were different, enabling biological inferences. The interpretation of our findings indicates that band formation in *P*. *generosa*, a species with temperate affinity [18], has a slow and continuous increment, reaching its maximum growth in 6 months, and its shape is more similar to the theoretical sawtooth shape of the MIR progression in an individual. Conversely, for *P*. *globosa*, distributed in subtropical environments [53], band formation showed two phases. The first was a rapid increment in the growth band, whereas in the second phase, there was a broad stabilization period without increments in the growth band before the deposition of a new growth band. These differences in shell deposition were not identified when only a sinusoidal symmetrical model was applied to the MIR data [8]. As mentioned before, this could be indicative of the degree of asynchrony in the formation of the growth increments, where this asynchrony would be stronger between the individuals of the subtropical population of *P*. *globosa* than in the temperate population of *P*. *generosa*. The difference in the trajectories described by the H2 model suggests the presence of particular factors that influence the formation of the growth band in each species, such as temperature [39,54] and food availability [55]. Generally, it has been observed in tropical regions that the variance of the data due to the lack of synchronicity in the formation of growth bands makes it difficult to detect a cycle or pattern (e.g., [56,57]); however, even in temperate species, the reading quality due to intrinsic characteristics of the hard structure and/or processing technique may preclude the detection of a pattern [58].

### Variability in growth increments

In this study, the variability in growth increments was only based on the hard structure (shell), and the main assumption was that the proportion of the growth increments were similar in all age structures of the population. Therefore, the growth increments in young and old individuals are deposited in the same proportion; under this condition, our results showed that for both species, there was no linear tendency of the growth increments through their lifespan. In this regard, Okamura et al. [12] used a simulation method to analyze the MIA data. Their method allowed them to analyze the individual variation in the timing of growth band formation and in the duration of the growth band after it is formed. Individual variation in the timing of growth band formation was controlled using the parameters in their model, expressing the individual variation in the duration of the growth band. Explicitly, Okamura et al. [12] analyzed the individual variability, which is useful and important to confirm that the estimated periodicity of growth band formation is invariant in the hard structures, and it is independent of the size of the individuals. Furthermore, Okamura et al. [12] recognized that their method would fail to identify the correct periodicity of the MIR if there are missed growth bands in the otoliths, a problem that, according to them, generally occurs in old fishes. Explicitly, our study did not analyze the age effect. According to Morita and Matsuishi [59], the concept “age effect” refers to a conservative process in which a hard structure (in such a case, the literature refers mainly to otoliths) increases in size continuously, even during periods of starvation or negative somatic growth, due to the physicochemical and obligatory microincrementation processes involved in the daily physiological cycle [60]. Thus, fish growth and hard structure (e.g., otolith) growth could be uncoupled. Therefore, a specific analysis of the “age effect” involves growth in hard structures associated with somatic growth in individuals studied (relationship between fish length and hard structure size). From the measures of the MIR reported in our study, only the shell (hard structure) was used to obtain the marginal increment data, and the somatic growth data for geoducks were not used in our analysis because they are not necessary. Consequently, for both species, we confirm that there was no linear tendency of the growth increments for *P*. *generosa* and *P*. *globosa* throughout their lifespan.

## Conclusion

In this study, we proposed a modification of the analysis and interpretation of the MIR by confirming that the proportion of the growth increments were similar in all age structures in populations of both *P*. *generosa* and *P*. *globosa*, and we defined the temporal periodicity of growth increments according to two harmonic functions through a multi-model analysis. On the basis of the parameters estimated in the models, this procedure enables us to generate information regarding the duration and seasonality of the period of growth mark formation. Our proposed methodology allows us to improve the validation processes by virtue of the following attributes: (1) incorporation of the totality of the observed data without transformation, even anomalous data, in the analysis; (2) identification of whether the data follow a cyclical pattern, and if this is regular or irregular; and (3) provision of the most important result in an age validation study, which is used to estimate the time of formation of the growth increments. This proposed age validation method was successfully tested by applying it to the growth increments of the shells of two clam species; however, we believe that it can be applied to different types of hard structures where MIR can be measured.

## Supporting information

S1 Table Datasets. Sample ID as well as its month and year of capture, corresponding MIR percentage (MIR %) and estimated age for both *P*. *generosa* from Punta Canoas (Table A) and *P*. *globosa* from Bahía Magdalena (Table B).
(XLSX)
